# NF-κB signaling is essential for resistance to heat stress-induced early stage apoptosis in human umbilical vein endothelial cells

**DOI:** 10.1038/srep13547

**Published:** 2015-09-04

**Authors:** Yanan Liu, Gengbiao Zhou, Zhenglian Wang, Xiaohua Guo, Qiulin Xu, Qiaobing Huang, Lei Su

**Affiliations:** 1 Southern Medical University, Guangzhou, China; 2 Guangzhou University of Chinese Medicine, Guangzhou, China; 3 Department of Pathophysiology, Southern Medical University, Guangzhou, China; 4 Department of ICU, General Hospital of Guangzhou Military Command, Key Laboratory of Tropical Zone Trauma Care and Tissue Repair of PLA, Guangzhou, China; 5 Postdoctoral Workstation, Huabo Bio-pharmaceutical Research Institute, Guangzhou, China

## Abstract

Cell apoptosis induced by heat stress is regulated by a complex signaling network. We previously reported that a p53-dependent pathway is involved. Here, we present evidence that NF-κB signaling plays a crucial role in preventing heat stress-induced early apoptosis. Human umbilical vein endothelial cells (HUVECs) were examined and increased phosphorylation of p65 and IκBα were detected, without IκBα degradation. When NF-κB signaling was inhibited by BAY11-7082, or a small interference RNA (siRNA) targeting *p65*, a significant increase in cell apoptosis and caspase-3 activity was observed, as well as reduced expression and translocation of HSP27 into the nucleus, an accumulation of reactive oxygen species, and prolonged phosphorylation of mitogen-activated protein kinases (MAPKs). In addition, an association between HSP27 and p65 was identified which may enhance NF-κB activation. When HSP27 was overexpressed, pretreatment of HUVECs with the antioxidant, apocynin, or N-acetyl cysteine, suppressed apoptosis. Similarly, inhibition of JNK and p38 with SP600125 and SB203580, respectively, also suppressed apoptosis, whereas siRNA-mediated *HSP27* knockdown and treatment with the ERK 1/2 inhibitor PD98059 did otherwise. In conclusion, these findings suggest a novel role for an NF-κB signaling pathway involving HSP27, ROS, and MAPKs that confers a protective effect against heat stress-induced cell apoptosis.

Heatstroke is a life-threatening condition that typically develops following exposure to extended periods of high temperatures. It is characterized by a rapid increase in core temperature to more than 40 °C and multiple organ dysfunction syndrome (MODS)^1^,^2^,^3^. The critical maximum temperature for the human body is between 41.6 °C and 42.0 °C. Previous studies have suggested that apoptosis is a major cause of cell death in heatstroke, and that it can be induced within a few hours^4^,^5^. It is hypothesized that endothelial cell activation/injury contributes to the pathophysiology of heat stroke^6^, and endothelial damage has been detected in heatstroke patients^7^,^8^. In addition, recent studies have reported that the acute phase of heat stress induces significant apoptosis in endothelial cells^9^, and we recently reported that intense heat stress induces early apoptosis via a transcription-independent mitochondrial p53 pathway^10^. However, the mechanisms mediating cell death in the late phase of heat stress remain unclear.

NF-κB is an important intracellular signaling protein that controls the transcription of several genes involved in cell growth, inflammatory responses, cell survival, and cell apoptosis^11^. When NF-κB is associated with inhibitory molecules of the IκB family in the cytosol, it is inactive. Correspondingly, most of the inducers that activate NF-κB use a common pathway that involves phosphorylation-induced degradation of IκB proteins. The latter includes the major protein, IκBα, which was the first protein described for this family and is also the most extensively studied IκB protein to date^12^. Phosphorylation and degradation of IκBα requires phosphorylation of the upstream target, IκB kinase (IKK), which contains two catalytic subunits, IKKα and IKKβ^13^. Upon release from the NF-κB/IκBα dimer, NF-κB translocates from the cytoplasm into the nucleus to bind DNA and regulate transcription.

The NF-κB signaling pathway has a critical role in regulating various aspects of the apoptotic program^14^. For example, NF-κB activation has been shown to down-regulate pro-apoptotic JNK signaling in many cell types, thereby preventing apoptosis^15^,^16^. However, in certain pathological conditions, such as ischemia, the excessive accumulation of reactive oxygen species (ROS) can induce apoptosis or necrosis by activating mitogen-activated protein kinase (MAPK) and caspase signaling cascades, and/or by disrupting mitochondrial membrane potential in Jurkat and in HeLa cells^17^. NF-κB has also been shown to exert pro-survival functions by inhibiting TNF-α-induced ROS accumulation-mediated prolongation of MAPK activation and necrotic cell death in murine embryonic fibroblasts^18^. Despite these insights, however, it remains unknown whether ROS play a critical role in heat stress-induced MAPK activation, and whether NF-κB has a role in mediating oxidative stress and MAPK signaling pathways under physiological conditions in HUVECs.

Heat shock proteins (HSPs) are an evolutionarily conserved set of proteins that mediate a cell’s response to heat stress, and a subset of HSPs protects cells against an induction of cell death (including apoptosis and necrosis) in response to a variety of stresses^19^. In particular, HSP27 and HSP70 have been shown to contribute to the regulation of NF-κB activation in many different cell types^20^,^21^,^22^,^23^,^24^, with a direct link observed between HSP27 and regulation of the NF-κB signaling pathway in cell apoptosis. For example, in macrophage-conditioned intestinal epithelial cells stimulated with interleukin-1β (IL-1β), HSP27 was shown to bind and suppress IKK to regulate NF-κB activation^25^. Similar mechanisms have been found in keratinocytes stimulated with tumor necrosis factor-α (TNF-α) and UV irradiation^26^, and in HeLa cells stimulated with TNF-α^20^. Moreover, when HSP27 was overexpressed in response to various stimuli, it facilitated proteasome-mediated proteolysis via phosphorylated IκBα and enhanced NF-kB activity^27^. The latter observation is consistent with the antiapoptotic properties reported for HSP27^27^. To investigate whether regulation of NF-kB activation by HSP27 affects heat stress-induced cell apoptosis, various experiments were performed using HUVECs as a model. As a result, a novel NF-κB signaling pathway was identified that includes HSP27 protein expression and translocation into the nucleus, the accumulation of ROS, and subsequent MAPK activation.

## Results

### Heat stress activates NF-κB during the recovery period following heat stress

**Figure 1:**
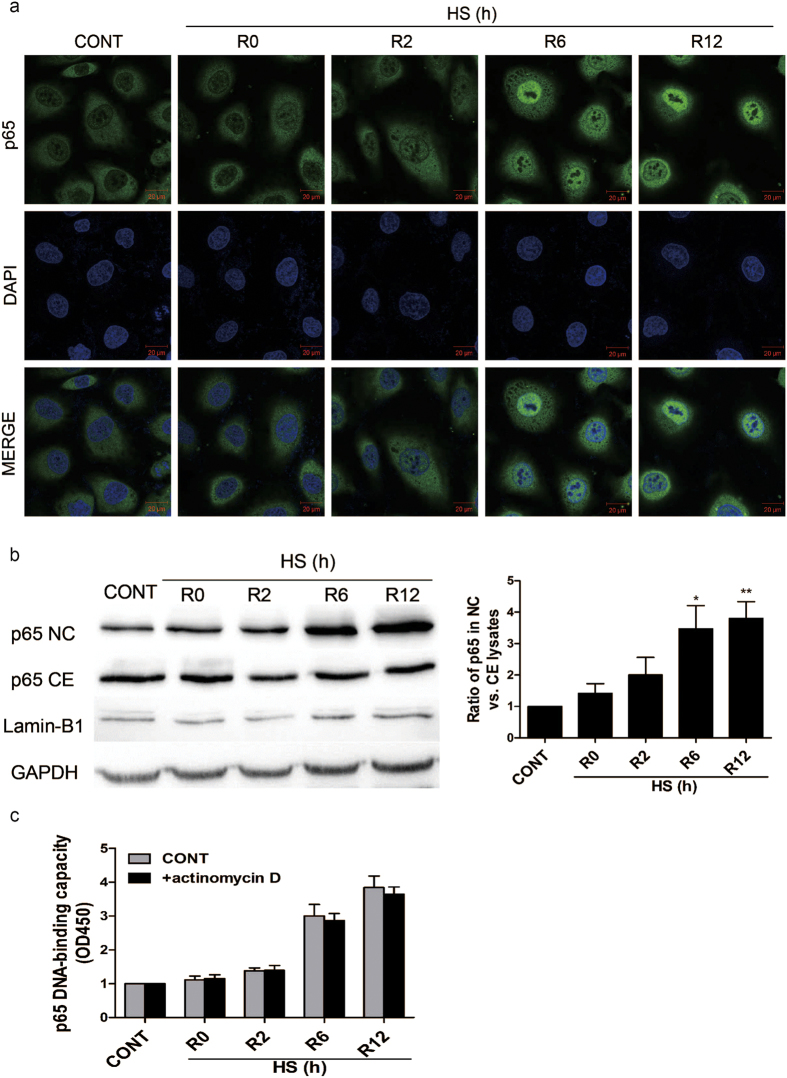
Relocalization of p65 from the cytosol into the nucleus of heat stressed HUVECs. Cells were incubated at 37 °C (CONT) or were subjected to a heat stress (HS) treatment at 43 °C for 90 min, followed by a recovery period at 37 °C for 0 h (R0), 2 h (R2), 6 h (R6), or 12 h (R12). (**a**) The cells were then fixed and processed for indirect immunofluorescence analysis using an antibody raised against the p65 subunit of NF-κB. Representative images are shown. (**b**) Expression of p65 was detected in cytoplasmic (CE) and nuclear (NC) fractions of HUVECs by Western blotting. The cropped images represent blotting experiments that were performed under the same experimental conditions. (**c**) HUVECs were pretreated with or without 0.5 μg/ml actinomycin D 5 min before being incubated at 37 °C (CONT) or being subjected a HS treatment. Whole cell extracts were prepared and NF-κB binding to DNA was quantified with the Trans-AM^TM^p65 transcription factor assay kit. Each value represents the mean ± SD of three independent experiments. **P < 0.01 and ***P < 0.001 versus control.

The transcription factor, NF-κB, has been shown to be activated during the recovery period following heat stress in HeLa cells^28^. Therefore, in this study, it was investigated whether heat stress activates NF-κB in human umbilical vein endothelial cells (HUVECs). After HUVEC cells were grown in culture media for 48 h, the culture dishes were sealed with parafilm and immersed in a circulating water bath maintained at 43 °C to induce heat stress^29^. After 90 min, the culture media was replaced with fresh media and the cells were further incubated at 37 °C for various periods of time (e.g., 0, 2, 6, and 12 h) before being assayed. Indirect immunofluorescence studies demonstrated that the distribution of p65 in nucleus was obviously increased after 6–12 h of heat stress recovery at 37  °C (Fig. 1a). Then nuclear and cytoplasmic extracts of the same timepoints were collected and analyzed by western blot. The result revealed that, unexpectedly, the amount of p65 in nuclear and cytoplasm were both increased (Fig. 1b). It is possible that this increase of nucleus p65 is due to the increase of p65 synthesis, rather than the shuttling of p65 from cytoplasm to nucleus.

To address this issue, 0.5 μg/ml actinomycin D was added to HUVECs 5 min prior to the heat stress treatment in order to block transcription and translation^28^. An ELISA-based TransAM NF-κB Activation kit was then used to quantify NF-κB binding to DNA^30^,^31^. Pretreatment with actinomycin D did not modify the DNA-binding kinetics of NF-κB during the heat stress recovery period (Fig. 1c), thereby suggesting that no new translational events are required during heat stress recovery for NF-κB activation. Taken together, these data suggest that heat stress induces the translocation and activation of NF-κB during the recovery period in HUVECs.

### NF-κB activation during heat stress recovery occurs with prior phosphorylation, and without degradation of the IκB subunits

**Figure 2:**
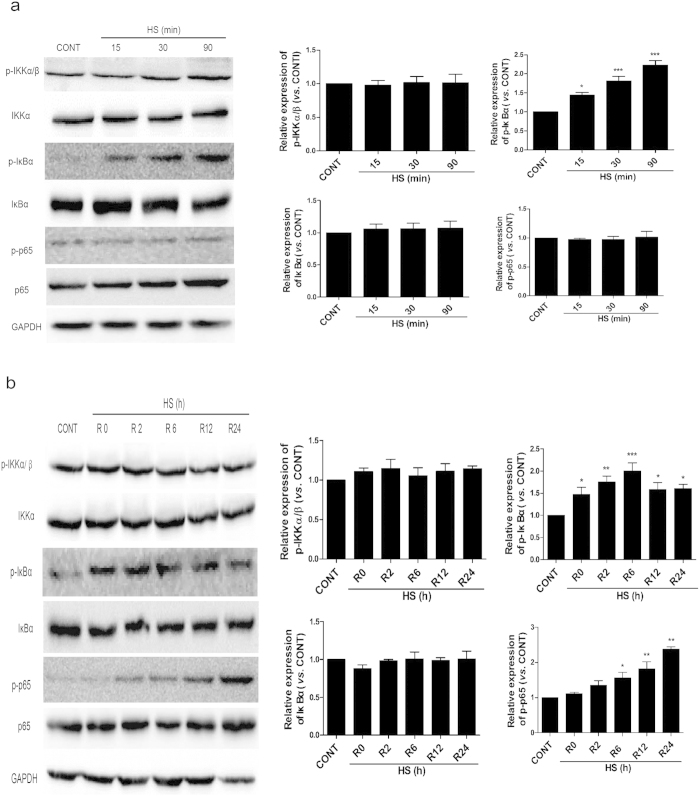
NF-κB activation during the heat stress recovery period involves the phosphorylation of IκBα and p65, without degradation of IκBα. HUVECs were either maintained at 37 °C (CONT) or were subjected to a heat stress treatment (HS) at 43 °C for 15, 30, or 90 min (**a**), or a HS at 43 °C for 90 min, followed by a recovery period at 37 °C for 0 h (R0), 2 h (R2), 6 h (R6), 12 h (R12), or 24 h (R24) (**b**). Protein expression levels of IκBα, p-IκBα, p65, p-p65, p-IKKα/β, IKKα, and GAPDH for each set of samples were determined by Western blotting. The cropped images represent blotting experiments that were performed under the same experimental conditions. Each value represents the mean ± SD of three independent experiments. *P < 0.05, **P < 0.01, ***P < 0.001 versus control.

To determine whether activation of NF-κB is associated with the phosphorylation of IκBα, IKK-α/β, and/or p65, as well as the degradation of IκBα, Western blot assays were performed for HUVEC extracts collected during heat stress treatment (Fig. 2a) and during the recovery period following the heat stress treatment (Fig. 2b). Phosphorylation levels of IKK-α/β were high at 37 °C, and were unchanged by heat stress. In contrast, levels of phosphorylated IκBα and p65 were low at 37 °C, but increased after 15 min and 90 min of heat treatment (Fig. 2a,b). The levels of phosphorylated IκBα and p65 then further increased 24 h after the heat stress treatment was completed.

There were no signs of IκBα degradation observed during the heat stress treatment or during the recovery periods assayed. Thus, it appears that NF-κB activation is accompanied by phosphorylation of p65 and IκBα, and not by degradation of IκBα in HUVECs.

### NF-κB activation decreases heat stress-induced apoptosis

**Figure 3:**
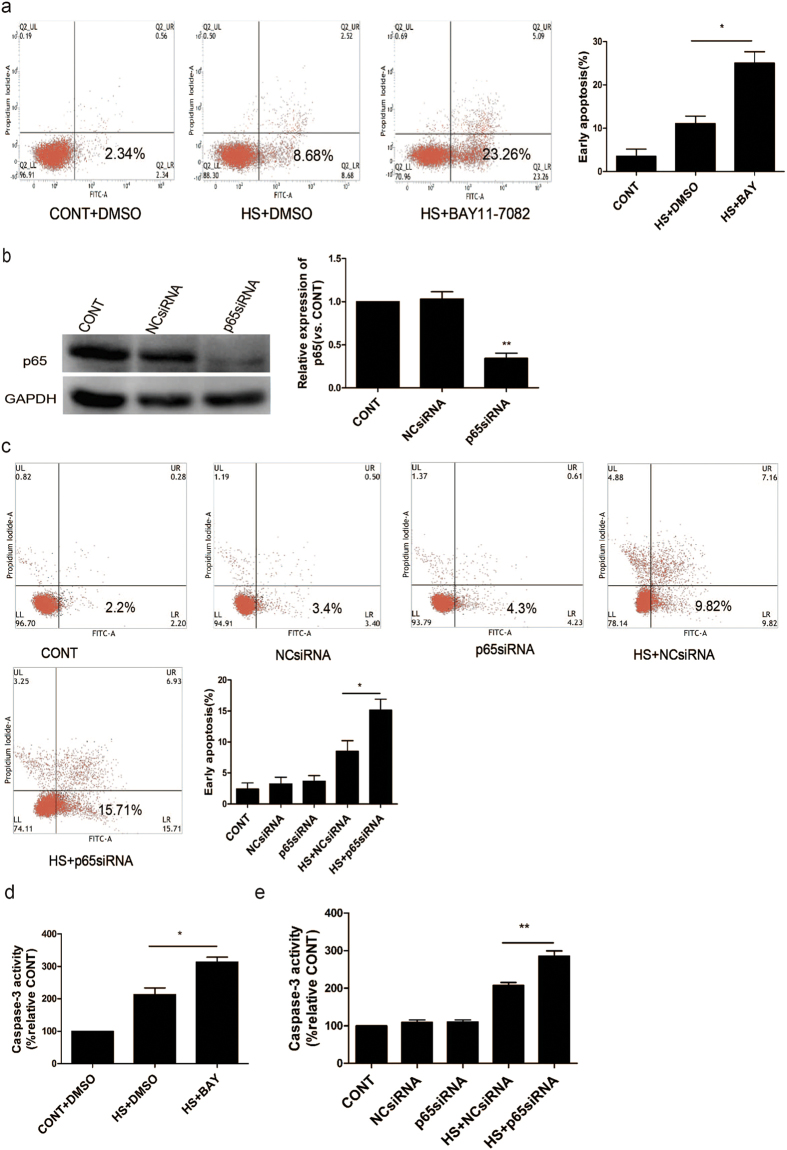
Influence of NF-κB on endothelial cell apoptosis induced by heat stress. (**a**) Cells were pretreated with DMSO or 5 μM BAY11-7082 (BAY) for 1 h, then were incubated at 37 °C (CONT) or 43 °C for 90 min (HS), followed by a recovery period at 37 °C for 24 h. Levels of apoptosis were detected with Annexin V-FITC/PI staining and were analyzed by flow cytometry. Levels of early apoptosis are shown in the bar graph at the far right. (**b**) HUVECs were transfected with a negative control siRNA (NCsiRNA) or a *p65*-targeted siRNA (p65siRNA) for 48 h. Protein expression of p65 was detected by Western blot and band density was quantified relative to GAPDH and is graphed at the right. (**c**) Apoptosis was detected for the samples characterized in (**b**) following staining with Annexin V-FITC/PI. Levels of early apoptosis were analyzed and are quantitated in the bar graph. (**d**) Enzymatic activity of caspase-3 was measured for the cell lysates indicated using the fluorogenic substrate, Ac-DEVD-AMC. Data are expressed as a relative increase to the control (CONT) and represent the mean ± SD of three independent experiments. *P < 0.05, **P < 0.01.

To investigate whether NF-κB activation affects heat stress-induced apoptosis, Annexin V-FITC/PI staining was used to differentiate early apoptosis from late apoptosis and necrosis. HUVECs that were pretreated with the NF-κB inhibitor, BAY11-7082, prior to heat stress treatment were more susceptible to high levels of early apoptosis compared with the HUVECs that were pretreated with DMSO (Fig. 3a). When HUVECs were transfected with a *p65*-targeted siRNA, lower levels of p65 protein were detected (Fig. 3b), and these cells were more susceptible to heat stress-induced early apoptosis (Fig. 3c).

Caspase-3 activity was also assayed for HUVECs that were pretreated with DMSO or the NF-κB inhibitor, BAY11-7082 (5 μM), for 1 h, and for HUVECs that were transfected with p65-targeted siRNA for 48 h, then underwent a heat stress treatment (Fig. 3d,e). Higher levels of caspase-3 activity were detected when NF-κB was inhibited and when levels of p65 were knocked down. Taken together, these results indicate that activation of NF-κB is essential for HUVECs to resist heat stress-induced apoptosis.

### Role of HSP27 in activating heat stress-induced apoptosis in HUVECs

**Figure 4:**
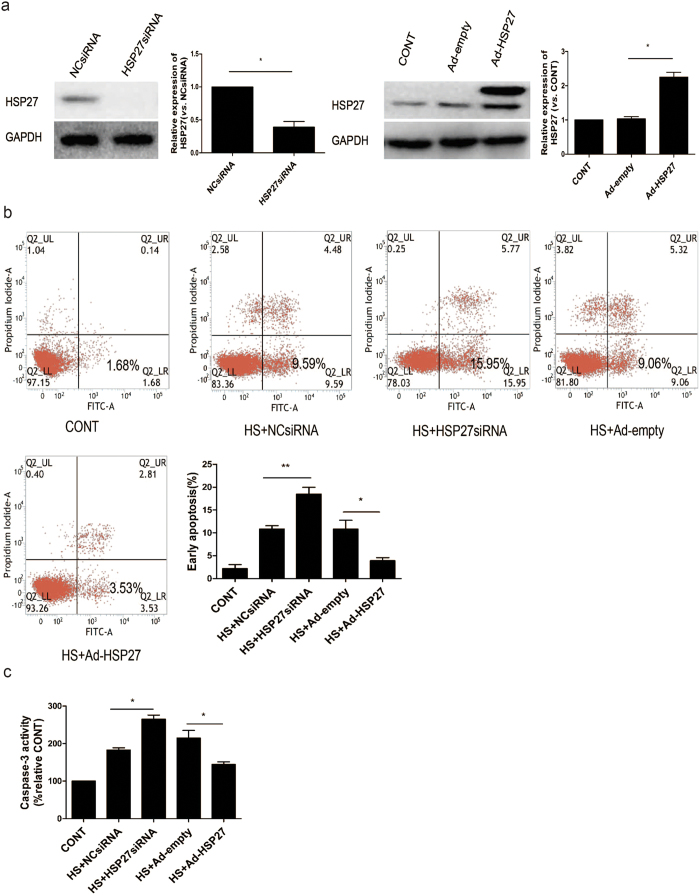
Role of HSP27 in heat stress-induced apoptosis in HUVECs. (**a**) Knockdown and overexpression of HSP27 were achieved in HUVECs following the transfection of: a negative control siRNA (NCsiRNA) and an *Hsp27*-targeted siRNA (HSP27siRNA), and an empty adenovirus vector (Ad-empty) and a adenovirus vector expressing HSP27 (Ad-HSP27), respectively. As shown in the Western blot analyses performed. The cropped images represent blotting experiments that were performed under the same experimental conditions. Expression levels were also quantitated relative to GAPDH. (**b**) Transfected HUVECs described in (**a**) were incubated at 37 °C (CONT) or were subjected to a heat stress (HS) treatment at 43 °C for 90 min, followed by a recovery period at 37 °C for 24 h. Apoptosis was analyzed by flow cytometry using Annexin V-FITC/PI staining. Levels of early apoptosis are shown in the bar graph. (**c**) Enzymatic activity of caspase-3 was measured for the cell lysates indicated using the fluorogenic substrate, Ac-DEVD-AMC. Data are expressed as relative activity to the control at 37 °C (100%). Each value represents the mean ± SD of three independent experiments. *P < 0.05, **P < 0.01.

To investigate the role of HSP27 in heat stress-induced cell apoptosis, HUVECs were transfected with an HSP27-targeted siRNA or an adenovirus expressing HSP27, and then were subjected to a heat stress treatment followed by a 24 h recovery period. The knockdown and overexpression of HSP27 that was initially achieved was detected in Western blot assays (Fig. 4a). Levels of apoptosis were subsequently analyzed by flow cytometry using Annexin V-FITC/PI staining. Higher levels of heat stress-induced apoptosis were detected in HUVECs following the knockdown of HSP27, while an increase in apoptosis levels were detected in cells overexpressing HSP27 (Fig. 4b). A similar profile was obtained when the corresponding cell lysates were analyzed for caspase-3 activity (Fig. 4c). In combination, these data suggest that HSP27 protects HUVECs from heat stress-induced apoptosis.

### Co-localization and functional correlation of HSP27 with NF-κB in HUVECs during the recovery period following heat stress

**Figure 5:**
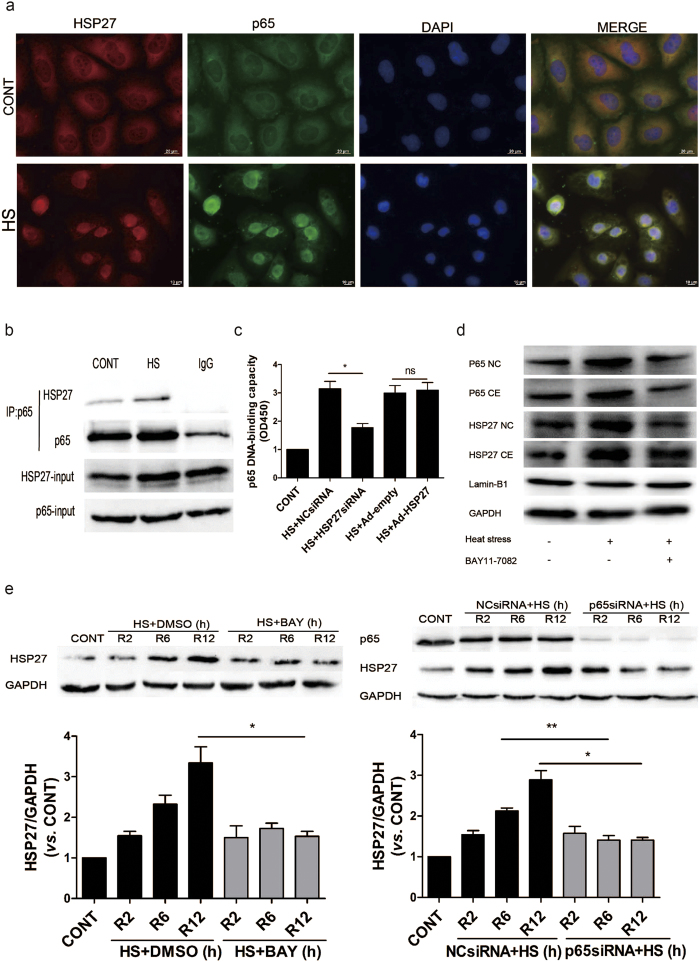
HSP27 associates and interacts with NF-κB in heat stressed HUVECs. HUVECs were incubated at 37 °C (CONT) or were subjected to a heat stress (HS) treatment at 43 °C for 90 min, then were further incubated for 6 h at 37 °C. (**a**) The cells were subsequently stained with an anti-HSP27 antibody (shown in green) and an anti-p65 antibody (shown in red). Co-staining with DAPI was used to visualize the nuclei (shown in blue). Merged images of these three stainings are shown in the right panels. (**b**) Whole cell lysates were prepared and probed in co-immunoprecipitation assays with an anti-p65 antibody, while a normal IgG antibody was used as a negative control. Western blot assays were then performed for the immunoprecipitated samples with an anti-HSP27 antibody. (**c**) HUVECs were transfected with a negative control siRNA (NCsiRNA) or an *Hsp27*-targeted siRNA (HSP27siRNA), or with an empty adenovirus vector (Ad-empty) and a adenovirus vector expressing HSP27 (Ad-HSP27), respectively. These transfected cells were then incubated at 37 °C (CONT) or were subjected to a heat stress (HS) treatment at 43 °C for 90 min, and recovery period at 37 °C for 6 h. Binding of NF-κB to DNA was quantified by using a Trans-AM^TM^p65 transcription factor assay kit. The values represent the relative binding that was measured at 450 nm. (**d**–**e**) HUVECs were pretreated with DMSO or 5 μM BAY11-7082 for 1 h (**d**), or were transfected with a negative control siRNA (NCsiRNA) or a p65-targeted siRNA (p65siRNA) for 48 h (**e**). Then both sets of cells were incubated at 37 °C (CONT) or 43 °C for 90 min (HS), followed by recovery period at 37 °C for 2 h (R2), 6 h (R6), or 12 h (R12). Expression levels of p65 and HSP27 were detected in Western blot assays performed. The cropped images represent blotting experiments that were performed under the same experimental conditions. Each value represents the mean ± SD of three independent experiments. *P < 0.05, **P < 0.01, ns: no significance. CE: cytoplasmic; NC: nuclear.

Based on the anti-apoptotic properties of HSP27 during heat stress recovery, and previous observations that HSP27 and NF-κB translocate into the nucleus in response to heat stress^28^,^32^, we hypothesized that NF-κB activation after heat stress may be linked to HSP27. Therefore, immunofluorescence studies of heat-stressed HUVECs were performed to detect the expression and localization of HSP27 and p65. Heat stress was found to stimulate the translocation of both HSP27 and NF-κB from the cytoplasm into the nucleus (Fig. 5a). To investigate the potential for interactions between NF-κB and HSP27, HUVECs were subjected to a heat stress treatment, they recovered at 37 C for 6 h, and then whole cell lysates were prepared. Subsequent co-immunoprecipitation assays were performed with an anti-NF-κB p65 antibody and Western blotting revealed that NF-κB p65 co-immunoprecipitated with HSP27 both at 37 °C and 6 h after a heat stress treatment (Fig. 5b).

To further assess the interactions between NF-κB and HSP27, HUVECs were transfected with an HSP27-targeted siRNA or an adenovirus expressing HSP27, and then were subjected to a heat stress treatment followed by a 6 h recovery period. Following the knockdown of HSP27 expression, DNA binding by NF-κB in the nucleus decreased (Fig. 5c). Although the NF-κB DNA-binding capacity did not notably increase after HSP27 overexpression, it may be that the levels of HSP27 themselves were increased after heat stress treatment. These results suggest that HSP27 may facilitate the nuclear import of NF-κB.

Meanwhile, blocking NF-κB activation by the inhibitor BAY11-7082 also prevents Hsp27 from going to the nucleus (Fig. 5d). However, when the HUVECs were pretreated with BAY11-7082 before heat treatment, the nuclear and cytoplasmic levels of p65 decreased. It is possible that a feedback loop exists whereby NF-κB regulates p65 expression. Moreover, our data showed that heat stress significantly increased HSP27 protein expression during heat recovery periods, which were decreased by inaction of NF-κB with BAY11-7082 or p65siRNA (Fig. 5e). These results suggested that NF-κB signaling regulates the translocation and expression of HSP27.

### Influence of NF-κB signaling on MAPK activation

**Figure 6:**
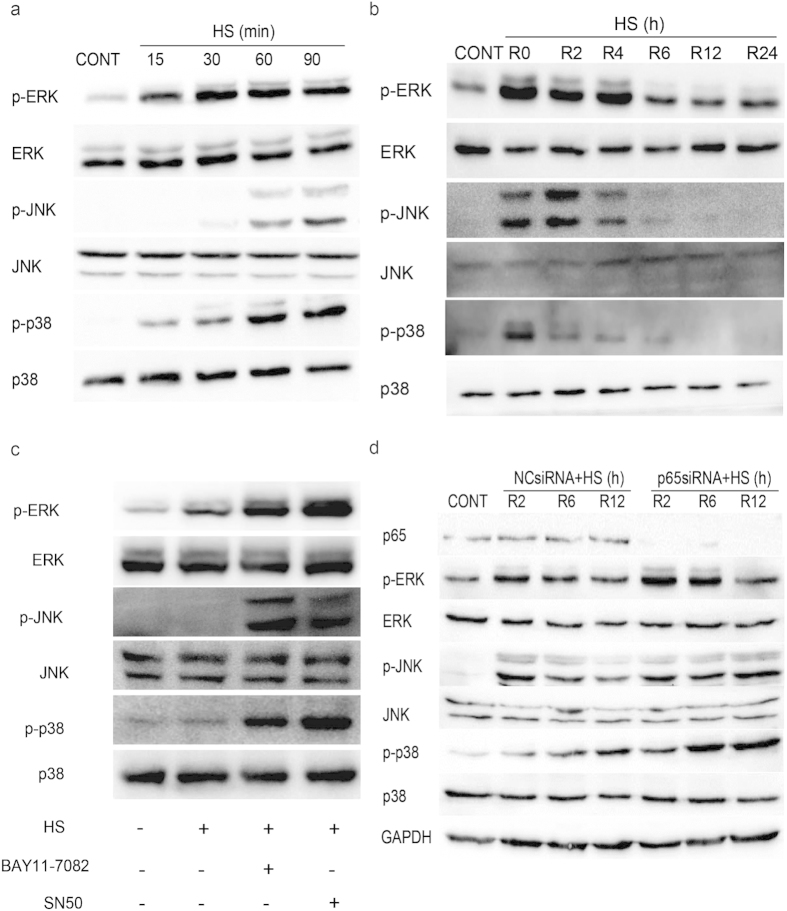
Prolonged MAPK activation detected in heat stressed HUVECs. (**a**) HUVECs were incubated at 37 °C (CONT) or were subjected to heat stress (HS) treatments at 43 °C for the indicated times. (**b**) HUVECs were incubated at 37 °C (CONT) or were subjected to a heat stress treatment at 43 °C for 90 min, and then were further incubated at 37 °C for the indicated times. (**c**) HUVECs were treated with BAY11-7082 or SN50 for 1 h prior to a heat stress treatment at 43 °C for 90 min. The cells were then further incubated at 37 °C for 6 h. (**d**) HUVECs were transfected with a negative control siRNA (NCsiRNA) or a p65-targeted siRNA (p65siRNA) and were incubated at 37 °C (CONT) or were subjected to a heat stress (HS) treatment at 43 °C for 90 min, followed by a recovery period at 37 °C for the indicated times. For a-d, the lysates were blotted with antibodies specific for activated forms of JNK (phospho-JNK), p38 (phospho-p38), and ERK (phospho-ERK), with detection of GAPDH used as a loading control. The membranes were reblotted with antibodies to detect levels of total JNK, p38, and ERK. All of the cropped images represent blotting experiments that were performed under the same experimental conditions.

Extracellular signal-regulated kinase (ERK), c-Jun NH2-terminal kinase (JNK), and p38 are members of well-characterized subfamilies of MAPK, and these enzymes have been implicated in the increased sensitivity to heat stress-induced cell apoptosis exhibited by IEC-6 cells^33^. To examine the role of these three kinases in HUVECs during heat stress and after various periods of recovery following heat stress, HUVEC extracts were analyzed by Western blot. Phosphorylation of ERK and phosphorylation of p38 were significantly induced after 15 min at 43 °C, while phosphorylation of JNK occurred after 60 min at 43 °C (Fig. 6a). During the recovery period. MAPK phosphorylation was rapidly induced and was maintained at high levels for more than 4 h before decreasing to baseline levels after a 6 h recovery period (Fig. 6b). Since the decrease in MAPK levels temporally coincided with NF-κB activation, it was hypothesized that activation of NF-κB inhibited MAPK phosphorylation. Therefore, HUVECs were pretreated with BAY11-7082, SN50, or *p65*-targeted siRNA, then were subjected to a heat stress treatment and a 6 h recovery period (Fig. 6c,d). Decreasing levels of MAPK phosphorylation were observed except when NF-κB was inactivated. These data suggest that heat stress-induced phosphorylation of MAPK proteins is inhibited by NF-κB activation 6 h after a heat stress event.

### ROS mediates the inhibition of MAPK phosphorylation by NF-κB activation

**Figure 7:**
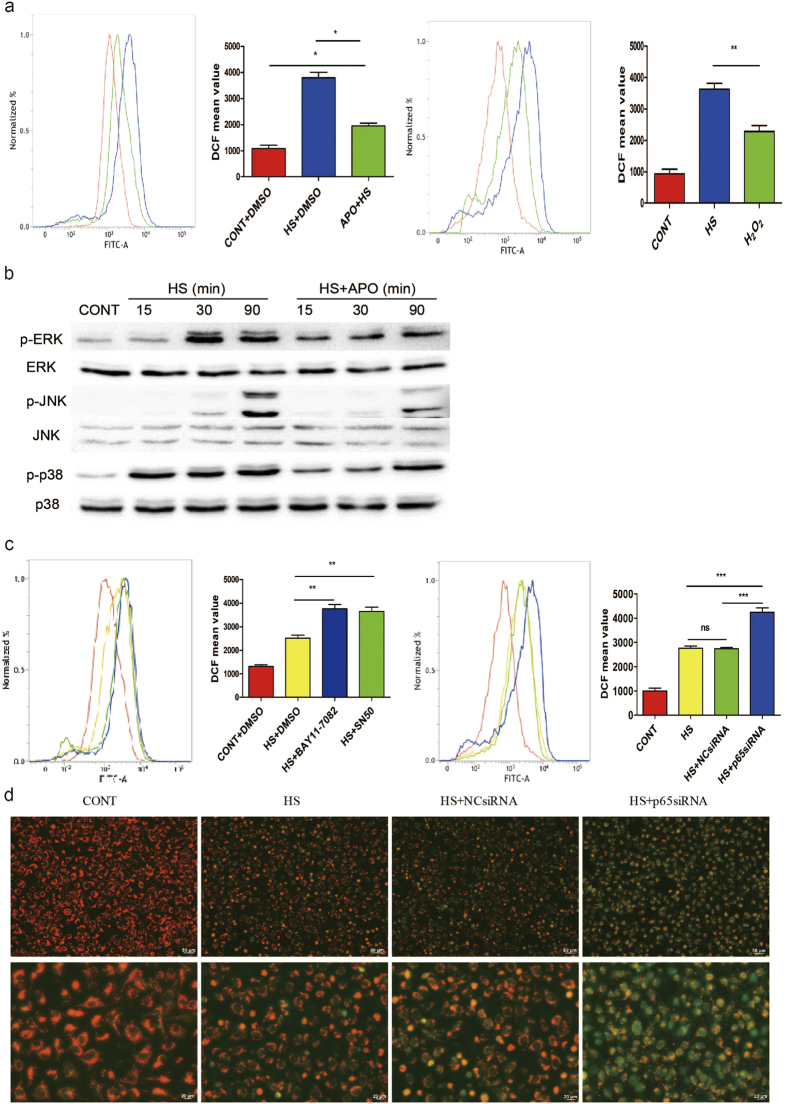
Inactivation of NF-κB induced the accumulation of ROS in HUVECs during the heat stress recovery period. (**a**) HUVECs were pretreated with DMSO or APO and then were subjected to 37 °C (CONT) or heat stress (HS) treatment for 90 min, followed by a second incubation at 37 °C for 6 h. HUVECs were treated with 1 mM H_2_O_2_ as a positive control for ROS. Treated cells were labeled with DCFH-DA for the last 30 min. Fluorescence was detected by flow cytometry. (**b**) HUVECs were untreated or pretreated with APO (250 μM) for 30 min, and then were incubated at 37 °C (CONT) or were subjected to a heat stress (HS) treatment at 43 °C for the indicated time periods. The corresponding cell lysates were analyzed as described in Fig. 6, and the cropped gels were run under the same experimental conditions. (**c**) HUVECs were pretreated with DMSO or the NF-κB inhibitors, BAY11-7082 and SN50, for 1 h, or were transfected with a negative control siRNA (NCsiRNA) or a p65-targeted siRNA (p65siRNA) for 48 h. These cells were subsequently incubated at 37 °C (CONT) or 43 °C (HS) for 90 min, followed by a recovery period of 6 h at 37 °C. The cells were labeled with DCFH-DA for the last 30 min and fluorescence was detected by flow cytometry. (**d**) HUVECs were transfected with NCsiRNA or p65siRNA for 48 h, then were incubated at 37 °C (CONT) or 43 °C (HS) for 90 min, followed by a recovery period at 37 °C for 6 h. Mitochondrial membrane potential (ΔΨm) was assessed with JC-1staining: red cells represent JC-1 aggregates due to an intact ΔΨm. Conversely, the green cells represent JC-1 monomers due to a disrupted ΔΨm. Each value represents the mean ± SD from three independent experiments. *P < 0.05, **P < 0.01, ***P < 0.001.

As shown in Fig. 7a, the antioxidant apocynin (APO) inhibited ROS accumulation in the heat stress group, but the effects were not complete in untreated HUVECs. We utilized the fluorescent dye DCFH-DA, which produces enhanced fluorescence when cells generate ROS, and analyzed fluorescent signals by flow cytometry. H_2_O_2_ was used as a positive control. Consistent with the partial inhibitory effect of APO on heat stress-induced ROS accumulation, APO only partially inhibited heat stress-induced MAPK activation (Fig. 7b). These results demonstrate that accumulation of ROS perfectly coincides with prolonged MAPK activation.

The observed inhibition of MAPK activation by APO treatment in HUVECs prompted us to examine whether heat stress stimulation induces ROS accumulation in BAY11-7082– and SN50-pretreated cells and p65-depleted HUVEC cells. A substantial increase in the fluorescent signals of DCFH-DA was observed in BAY11-7082– or SN50-pretreated cells during the heat stress recovery period compared to the unpretreated group. The same results were obtained with p65-depleted HUVECs (Fig. 7c). In addition, p65-depleted HUVEC cells significantly increased the heat stress-induced the loss of mitochondrial membrane potential (ΔΨm) (Fig. 7d), thereby suggesting that mitochondria may provide a permanent source of ROS in heat stressed HUVECs. Overall, the results of these experiments suggest that ROS contribute to the signaling pathway involving NF-κB activation-induced phosphorylation of MAPKs in response to heat stress.

### Contribution of ROS and MAPKs to heat stress-induced cell apoptosis in HUVECs

**Figure 8:**
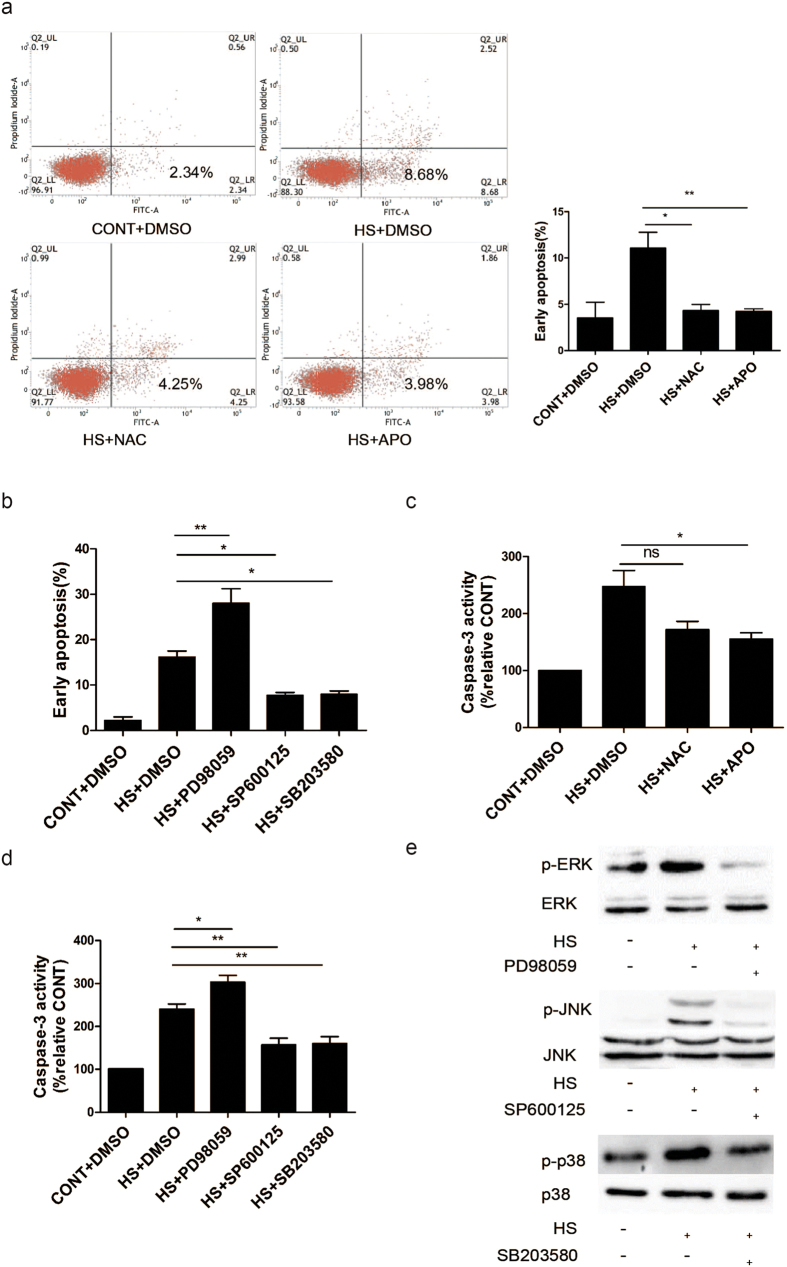
The role of ROS and MAPKs in heat stress induced cell apoptosis in HUVECs. (**a**) HUVECs were pretreated with DMSO or APO or NAC prior to being incubated at 37 °C (CONT) or 43 °C (HS) for 90 min, followed by a recovery period at 37 °C for 6 h. Apoptosis was analyzed by flow cytometry using Annexin V-FITC/PI staining. (**b**) HUVECs were pretreated with DMSO, PD98059, SP600125, and SB203580 (specific inhibitors of ERK, JNK, and p38, respectively), prior to being incubated at 37 °C (CONT) or 43 °C (HS) for 90 min, followed by a recovery period at 37 °C for 6 h. Apoptosis was analyzed by flow cytometry using Annexin V-FITC/PI staining and was quantitated. (**c**,**d**) Enzymatic activity of caspase-3 was detected for the cell lysates of the cells analyzed in panels A and B. Data are expressed as the fluorescence increase relative to the control at 37 °C. (**e**) HUVECs were pretreated with or without MAPK inhibitors (PD98059, SP600125, and SB203580, respectively) for 30 min prior to a heat stress (HS) treatment at 43 °C for 90 min. Cell lysates were subsequently collected and analyzed by Western blot as described in Fig. 6. The cropped gels were run under the same experimental conditions. Each value represents the mean ± SD of three independent experiments. *P < 0.05, **P < 0.01, ns: no significance.

To examine whether accumulated ROS or MAPK activation participates in heat stress-induced cell apoptosis, HUVECs were stimulated with heat stress in the presence or absence of inhibitors for ROS or MAPKs. As shown in Fig. 8a, treatment with APO or N-acetyl-L-cysteine (NAC) alone substantially decreased the number of cells undergoing early apoptosis. Furthermore, while HUVECs pretreated with PD98059, a specific inhibitor of ERK, exhibited an increase in cell apoptosis, HUVECs pretreated respectively with specific inhibitors of JNK and p38, SP600125 and SB203580, exhibited a significant decreases in cell early apoptosis (Fig. 8b). Caspase-3 activity was also assayed (Fig. 8c,d), and these results are consistent with the apoptosis data. Finally, We verified that these inhibitors actually inhibited MAP kinase activities by using antibodies specific for phosphorylated form of JNK, ERK, or p38 (Fig. 8e). The three MAPK inhibitors examined also did not affect the heat stress-induced accumulation of ROS in HUVECs (data not shown), thereby indicating that MAPK activation is a downstream event of ROS accumulation.

## Discussion

In this study, we demonstrate that activation of the NF-κB signaling pathway is essential for resistance to heat stress-induced apoptosis in HUVECs, and this pathway includes roles for NF-κB in mediating an increase in the expression and translocation of HSP27 into the nucleus, as well as an inhibition of ROS accumulation and subsequent MAPK activation. Together, these actions by NF-κB lead to its anti-apoptotic role in HUVECs. Moreover, the association between HSP27 and p65 was found to regulate the heat stress-induced activation of NF-κB that affects HUVEC survival.

In HeLa cells, it has been demonstrated that heat shock is a very powerful inducer of NF-κB-dependent transcription^28^. Accordingly, it has been hypothesized that hyperthermic stress as a result of heat stroke can lead to recruitment of NF-κB. However, in a previous study, binding of DNA by NF-κB was not detected in cells that underwent heat shock^34^,^35^. It has been proposed a mild heat treatment, or one of short duration, without a recovery period does not lead to NF-κB activation^28^. In the present study, NF-κB activation in HUVECs was not observed until 6 h after administration of a heat stress treatment. In addition, NF-κB was found to only bind DNA during the recovery period.

To date, there have been several exceptions to the universal pathway of NF-κB activation depending on the cell type and extracellular stimuli studied^14^. For example, activation of NF-κB in response to UV radiation or amino acid analog treatment requires IκBα degradation without prior phosphorylation^36^,^37^, while anoxia stimulates IκBα phosphorylation at Tyr42, leading to the release of p65/p50 from the NF-κB·IκBα complex^38^. NF-κB can also be activated by Ang II in vascular smooth muscle cells via phosphorylation of p65 at Ser536, and not via phosphorylation of IκBα^39^. In HeLa cells, NF-κB activation during the heat stress recovery period has been associated with the thermolability of the NF-κB·IκBα complex, independent of prior phosphorylation or degradation of the IκB subunits^28^. In the present study, a novel mechanism for heat stress-induced NF-κB activation is characterized in endothelial cells, and this mechanism involves phosphorylation of p65 and IκBα, without IκBα degradation, and this differs from the NF-κB activation pathway that has been characterized in HeLa cells.

In diverse cell types, NF-κB signaling has been shown to have a critical role in regulating the apoptotic program. Moreover, whether NF-κB promotes or inhibits apoptosis appears to depend on the cell type or inducer studied^14^. In the experiments performed in the present study to determine whether NF-κB activation plays a role in heat stress-induced apoptosis in HUVECs, a significant increase in the levels of early cell apoptosis were detected 24 h after the heat stress treatment. Furthermore, when NF-κB activation was inhibited by BAY11-7082 or *p65*-targeting siRNA, the levels of cell apoptosis markedly increased. Nivon *et al.*^40^ previously described a significant increase in late apoptosis and necrosis in HeLa cells during the recovery period following heat stress, and these levels were markedly reduced by NF-κB-activated autophagy. Interestingly, early apoptosis was the predominant event observed in the present study following heat treatment, while only a few late apoptosis and necrosis events were detected. This difference is attributed to the different cell types that were examined^33^.

Here, the anti-apoptotic effect of NF-κB was linked with HSP27, an important anti-apoptosis protein that translocates into the nucleus in response to heat stress. The findings of the present study also support the hypothesis that HSP27 facilitates the nuclear transport of NF-κB to regulate cell apoptosis. For example, HSP27 and NF-κB p65 exhibited similar cytoplasm-nucleus translocation events, HSP27 and NF-κB p65 were co-immunoprecipitated from heat stressed HUVEC lysates, and HSP27 and NF-κB p65 appeared to colocalize at 37 °C and 43 °C in HUVECs. In addition, knockdown of *HSP27* abolished NF-κB transactivation. Thus, attenuation of cell apoptosis by HSP27 is likely due, at least in part, to its enhancement of NF-κB activation.

There are many potential mechanisms by which HSP27 may regulate NF-κB activation given the broad range of cellular functions that HSP27 participates in. However, we hypothesize that HSP27 stabilizes and protects stress-labile NF-κB p65, thereby allowing for efficient translocation of NF-κB into the nucleus to modulate gene transcription and apoptosis. To our knowledge, the present study is the first to identify HSP27 as a positive regulator of the translocation of p65-associated NF-κB into the nucleus in response to heat stress. Furthermore, these results are in agreement with the data collected following the treatment of HUVECs with TNF-α, where HSP27 appeared to be essential for sustained NF-κB activation^41^. Meanwhile, NF-κB was found to increase HSP27 expression and its translocation into the nucleus since inhibition of NF-κB with BAY11-7082, or following depletion of *p65* with siRNA, led to a decrease in the expression and translocation of HSP27 during heat stress recovery. While additional studies are needed to determine the mechanism by which NF-κB modulates HSP27 expression, the results of the present study suggest that HSP27 is involved in regulating the NF-кB signaling pathway, and NF-кB functions upstream of HSP27 to regulate its transcription and nuclear accumulation. It is also possible that a positive feedback loop exists between HSP27 and the activation of NF-кB. Regardless, the data of the present study suggest that NF-κB/HSP27 signaling protects endothelial cells from heat stress-induced apoptosis.

The results of the present study are consistent with those from IEC-6 cells where heat stress was found to induce the accumulation of ROS and to reduce anti-oxidase activity and MAPK signaling to affect apoptosis^33^,^42^. Under physiological conditions, ROS are rapidly eliminated by antioxidant enzymes. However, the excessive accumulation of ROS can lead to the activation of MAPK and caspase signaling cascades, as well as the loss of ΔΨm, thereby leading to apoptotic or necrotic cell death^17^. In the present study, the accumulation of ROS significantly increased cell apoptosis and led to enhanced phosphorylation of ERK1/2, JNK, and p38. Moreover, these phosphorylation events were inhibited by activation of NF-κB, or the antioxidant, APO. These results suggest that NF-κB can inhibit heat-induced ROS accumulation that mediates MAPK activation. It is generally believed that ERKs are important for cell survival, while JNKs and p38 MAPKs have been characterized as stress-responsive factors, and thus, have a role in apoptosis^43^. Therefore, the dynamic balance between heat stress-activated ERK and JNK-p38 signaling pathways are hypothesized to be important for determining whether a cell survives or undergoes apoptosis. Here, heat stress-induced apoptosis was attenuated by the antioxidant, APO, or NAC, as well as by inhibitors of JNK (SP600125) and p38 (SB203580). In contrast, heat stress-induced apoptosis was exacerbated by inhibition of ERK1/2 by PD98059. Taken together, these data suggest that heat stress can induce both pro- and anti-apoptotic pathways via an NF-κB/ROS/MAPK signaling pathway in HUVECs. The most apparent effects under the conditions examined were anti-apoptotic.

**Figure 9:**
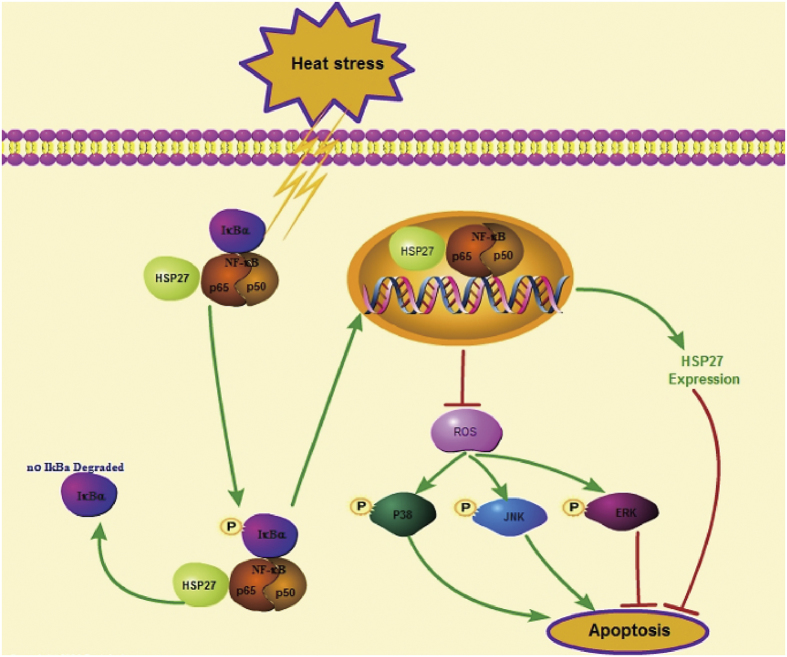
NF-κB activation during heat stress recovery follows a non-canonical signal transduction pathway in HUVECs with increased phosphorylation of p65 and IκBα, without IκBα degradation. Initially, heat stress stimulation induces NF-κB activation, and this leads to an anti-apoptosis effect involving inhibition of heat stress-induced ROS accumulation that normally mediates the activation of MAPKs. In heat-stressed HUVECs, ERK1/2 activation has an anti-apoptotic role, while activation of JNK and p38 is pro-apoptotic. The results of the present study confirm that HSP27 is an important anti-apoptotic protein, and NF-κB may modulate HSP27 expression levels and its translocation into the nucleus. In addition, HSP27 acting as a molecular chaperone may facilitate activation of this NF-κB pathway in HUVECs during heat stress recovery. Taken together, our findings suggest that this NF-κB signaling pathway involving HSP27, ROS, and MAPK is activated in response to heat stress and it confers a protective effect against heat stress-induced cell apoptosis.

In conclusion, the present study provides evidence that apoptotic cell death occurs in the late phase of heat stress recovery and it is regulated by a complex signaling network that is stimulated by NF-κB activation. This network also involves the expression and translocation of HSP27, ROS accumulation, and MAPK activation. Furthermore, the association of HSP27 with p65 appears to regulate heat stress-induced NF-κB activation (Fig. 9). Taken together, these results suggest that NF-κB signaling plays a novel role in the tolerance of cells to intense heat stress and it represents a potential therapeutic target for heat stroke.

## Methods

### Cell culture and treatments

HUVECs were purchased from the Shanghai Institute of Cell Biology, Chinese Academy of Sciences. Cells were grown in DMEM/F12 supplemented with 10% (v/v) fetal bovine serum (FBS), 100 U/ml of penicillin, and 100 μg/ml of streptomycin (Invitrogen Life Technology, USA) at 37 °C in a humidified atmosphere of 5% CO_2_ and 95% air. For the heat stress treatment, the bottom of each culture dish was placed into a circulating water bath that was maintained at 43 ± 0.5 °C for various periods of time as indicated. The cell culture media for each plate was then replaced with fresh media and the cells were further incubated at 37 °C as indicated. As a control, cell culture dishes were placed in a circulating water bath maintained at 37 ± 0.5 °C.

### Immunofluorescence analysis

Cell culture media was removed and cells were rinsed twice with PBS (2 min each) before being fixed in 3.5% paraformaldehyde and permeabilized in 0.1% Triton X-100 for 10 min at room temperature. After the cells were rinsed four times in PBS, the cells were incubated with PBS containing 5% FBS and 5% glycerol at 37 °C. After 45 min, the cells were incubated with a rabbit anti-human p65 antibody (1:50) or a mouse anti-HSP27 antibody (1:100) overnight at 4 °C. The cells were then washed with PBS twice (5 min each) and were incubated with an appropriate secondary antibody (1:100) at 37 °C. After 1 h, the cells were incubated with mounting solution containing 0.2 μg/ml DAPI for another 10 min. Images were acquired using a confocal fluorescence microscope.

### Western blot analysis

HUVECs were subjected to a heat stress treatment and then were lysed in NE-PER extraction reagent (Pierce) according to the manufacturer’s protocol to obtain cytoplasmic and nuclear protein extracts. Total protein concentrations were determined by the Bradford assay. Western blotting was performed as described previously^44^. The primary antibodies used included: p65 and HSP27 from Abcam (USA), and IκBα, p-IκBα, p-p65, p-IKKα/β, IKKα, p-JNK, p-p38, p-ERK, JNK, p38, ERK, and GAPDH from Cell Signaling Technology (USA). All of the antibodies were diluted 1:2000, and a horseradish peroxidase (HRP)-conjugated anti-rabbit IgG antibody was diluted 1:5000 as the secondary antibody (Zhongshan Inc., China). Signals were detected using enhanced chemiluminescence reagents (Pierce, USA).

### Measurement of NF-κB DNA-binding capacity by enzyme-linked immunosorbent assay (ELISA)

Nuclear extracts were prepared from treated and control HUVECs using a nuclear extraction kit (Active Motif, Inc). These extracts were then assayed for the ability of nuclear NF-κB p65 to bind a DNA consensus sequence provided by an ELISA-based TransAM NF-κB p65 kit (Active Motif), according to the manufacturer’s protocol.

### Analysis of cell apoptosis using Annexin V-FITC/PI staining and flow cytometry

Cell apoptosis was analyzed with an Annexin V-FITC apoptosis detection kit and flow cytometry according to the manufacturer’s protocol (Invitrogen). Briefly, ~1 × 10^6^ cells were collected, washed in ice-cold PBS, and resuspended in binding buffer containing a suitable amount of Annexin V-FITC. After a 10 min incubation at room temperature, the buffer was removed by centrifugation and the cells were resuspended in a reaction buffer containing propidium iodide (PI). After 10 min, a flow cytometric analysis was performed for each sample to detect apoptosis.

### Caspase-3 activity assay

After a heat stress treatment at 43 °C for 90 min, HUVECs were harvested and cell lysates were prepared at −80 °C for 30 min. The cells were then incubated with caspase-3 substrates at 37 °C and caspase-3 activity was measured based on cleavage of the fluorogenic peptide substrate^45^, Ac-DEVD-AMC, that was detected with a Quadruple Monochromator Microplate Reader (Infinite M1000, USA). Levels of caspase-3 activity are reported as relative cumulative fluorescence of the kinetic reaction compared to untreated controls.

### Transfection of siRNA

SiRNA targeting *p65* and *Hsp27* were designed and synthesized by GenePharma (Shanghai, China). The sequence of each gene and their corresponding negative controls are shown in Table 1. For transfection, HUVECs were plated onto 6-well plates (Nest, Biotech, China) at 30–50% confluence. Twenty-four hours later, HUVECs were incubated with siRNAMate Transfection Reagent (GenePharma Company, Shanghai, China) and siRNAs according to the manufacturer’s protocol. The transfected cells were collected 48–72 h later.

### Adenoviral infection

Adenoviruses (Ad) constitutively overexpressing HSP27 versus an empty control vector (Ad-empty) were constructed by ViGenen Biosciences (China). Cells were infected with the adenoviruses in serum-free DMEM for 6 h before the media was replaced with DMEM supplemented with 10% FBS.

### Co-immunoprecipitation (co-IP) assays

HUVEC cells were subjected to a heat treatment at 43 °C for 90 min, followed by an additional incubation period at 37 °C for the times indicated. Cell lysates (containing ~1.5 mg total protein) were subsequently harvested, and co-IP assays were performed by using the Thermo Scientific Pierce co-IP kit according to the manufacturer’s protocol.

### Measurement of ROS levels

Levels of intracellular ROS were assessed with a kit (Beyotime, Nanjing, China) that employs dichlorofluorescein diacetate (DCFH-DA, Molecular Probes, Beyotime) as a reagent that enters cells and reacts with ROS, thereby producing the fluorophore, dichlorofluorescein (DCF). Briefly, cells were maintained at 37 °C or were heated to 43 °C for 90 min, followed by a recovery period of various lengths at 37 °C. Approximately 3 × 10^5^ cells were then harvested, washed with serum-free DMEM culture medium, and stained with 1 μM DCFH-DA for 30 min at 37 °C in the dark. After the cells were centrifuged, washed, and resuspended in serum-free DMEM culture medium three times, the fluorescence intensity of each sample was determined with a flow cytometer.

### Mitochondrial membrane potential (ΔΨm) assay

The fluorescent dye, 5,5′,6,6′-tetrachloro-1, 1′,3,3′tetraethylbenzimidazolcarbocyanine iodide (JC-1; Molecular Probes, Eugene, OR, USA), was used to detect ΔΨm. Briefly, after a heat stress treatment, HUVECs were further incubated at 37 °C for various periods of time as indicated. After the cells were washed three times with PBS, a JC-1 kit and a fluorescence microscope were used to detect the mitochondrial cross membrane potential.

### Statistical analysis

All data were analyzed for statistical significance using SPSS 13.0 software (SPSS, USA). Data are expressed as the mean ± standard deviation (SD) from at least three independent experiments that were performed in duplicate. Statistical comparisons of the results were made using analysis of one-way ANOVA. *P < 0.05, **P < 0.01, and ***P < 0.001 were considered statistically significant.

## Additional Information

**How to cite this article**: Liu, Y. *et al.* NF-κB signaling is essential for resistance to heat stress-induced early stage apoptosis in human umbilical vein endothelial cells. *Sci. Rep.* **5**, 13547; doi: 10.1038/srep13547 (2015).

## Figures and Tables

**Table 1:** Oligonucleotide sequences used.

Target gene		Sequence (5′—3′)
p65	Sense	GCCCUAUCCCUUUACGUCATT
	Antisense	UGACGUAAAGGGAUAGGGCTT
HSP27	Sense	GUCUCAUCGGAUUUUGCAGC
	Antisense	GCUGCAAAAUCCGAUGAGACTT
Negative Control	Sense	UUCUCCGAACGUGUCACGUTT
	Antisense	ACGUGACACGUUCGGAGAATT
